# The future of lupin as a protein crop in Europe

**DOI:** 10.3389/fpls.2015.00705

**Published:** 2015-09-08

**Authors:** M. Mercedes Lucas, Frederick L. Stoddard, Paolo Annicchiarico, Juana Frías, Cristina Martínez-Villaluenga, Daniela Sussmann, Marcello Duranti, Alice Seger, Peter M. Zander, José J. Pueyo

**Affiliations:** ^1^Institute of Agricultural Sciences, ICA-CSIC, Madrid, Spain; ^2^Department of Food and Environmental Sciences, University of Helsinki, Helsinki, Finland; ^3^Research Centre for Fodder Crops and Dairy Productions, CRA, Lodi, Italy; ^4^Institute of Food Science Technology and Nutrition, ICTAN-CSIC, Madrid, Spain; ^5^Fraunhofer-Institute for Process Engineering and Packaging, Freising, Germany; ^6^Department of Food Environment and Nutritional Sciences, Università degli Studi di Milano, Milan, Italy; ^7^Terrena Lup’Ingredients, Martigne-Ferchaud, France; ^8^Leibniz Centre for Agricultural Landscape Research, ZALF, Müncheberg, Germany

**Keywords:** lupin, protein crop, plant protein, breeding, protein ingredients, protein foods, food supply chain

## Abstract

Europe has become heavily dependent on soya bean imports, entailing trade agreements and quality standards that do not satisfy the European citizen’s expectations. White, yellow, and narrow-leafed lupins are native European legumes that can become true alternatives to soya bean, given their elevated and high-quality protein content, potential health benefits, suitability for sustainable production, and acceptability to consumers. Nevertheless, lupin cultivation in Europe remains largely insufficient to guarantee a steady supply to the food industry, which in turn must innovate to produce attractive lupin-based protein-rich foods. Here, we address different aspects of the food supply chain that should be considered for lupin exploitation as a high-value protein source. Advanced breeding techniques are needed to provide new lupin varieties for socio-economically and environmentally sustainable cultivation. Novel processes should be optimized to obtain high-quality, safe lupin protein ingredients, and marketable foods need to be developed and offered to consumers. With such an integrated strategy, lupins can be established as an alternative protein crop, capable of promoting socio-economic growth and environmental benefits in Europe.

The demand of the ever-growing world population for dietary protein is no longer sustainable through animal products alone. Soya bean has become the prevalent source of plant proteins for food and feed, and Europe depends on soya bean imports for 70% of its plant protein requirements. White lupin (*Lupinus albus* L.), yellow lupin (*L. luteus* L.) and narrow-leafed lupin (*L. angustifolius* L.), are native European legumes that represent a significant alternative to soya bean. Their seed protein content is high (up to 44%) and its quality is good, they offer potential health benefits, and they contribute to the sustainability of cropping systems. Lupins are successful protein crops in Australia, where an important industry has developed to use lupin protein and other fractions, yet lupin production in Europe is insufficient to guarantee the stable and sufficient supply required for its use by the food and feed industry. Lupin is grown in several European countries, and although its grain yield is the world’s highest in some parts of Europe, its cropping area remains modest and yields are highly variable. A slight rise of European cultivated area and production has occurred during the period 2000–2013 (Figure 1), representing 17.6% of the world’s production during that period (Figure 2). The Andean lupin (*L. mutabilis* Sweet) has been brought into cultivation in some parts of South America, but is not present in Europe on a commercial scale.

**FIGURE 1:**
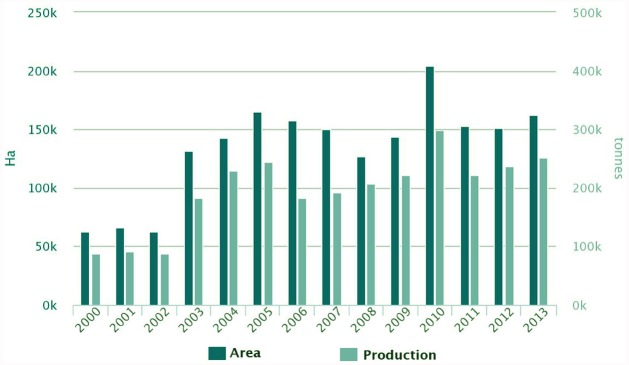
**Lupin cultivated areas and production in Europe.** Source: FAOSTAT 2015.

**FIGURE 2:**
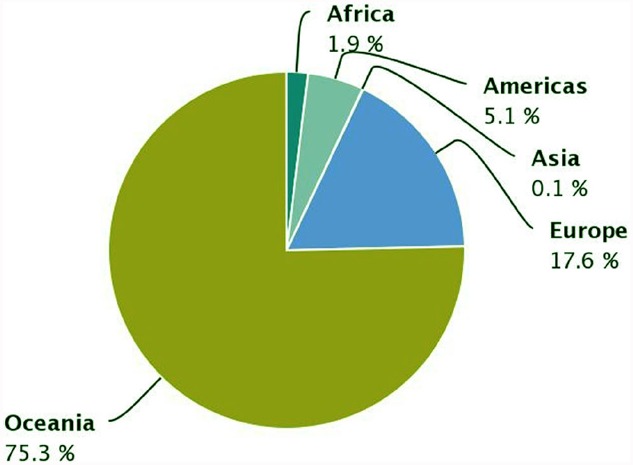
**Worldwide distribution of lupin production.** Source: FAOSTAT 2015.

Insufficient grain yield in certain areas, largely due to limited breeding, constitutes a significant barrier to the expansion and economic sustainability of lupin cultivation in Europe. Lupin breeding has exploited little of the gene pool (Berger et al., 2012), partly due to the need to maintain a low alkaloid content (sweet seed). Landrace germplasm offers tremendous opportunities for increasing white lupin yield (Annicchiarico et al., 2010). Lupins are relatively more tolerant to several abiotic stresses than other legumes, and have a proven potential for the recovery of poor and contaminated soils (Fernández-Pascual et al., 2007; Coba de la Peña and Pueyo, 2012). Identifying germplasm with tolerance to a range of abiotic stresses may allow lupin cultivation to expand into a wider range of agro-climatic conditions across Europe. In areas subjected to frequent or occasional frosts, high vernalization requirement along with intrinsic cold tolerance is required, as sudden winter frost may lead to high mortality even in regions with relatively mild winters (Annicchiarico and Iannucci, 2007). Summer drought and progressive soil salinization are major climatic stresses in Mediterranean areas, yet information is lacking on the intrinsic genetic variation for drought tolerance, particularly in white lupin. Poor adaptation to calcareous soils is probably the main limit to the crop expansion, but white lupin landraces from Egypt or Italy that display tolerance to free calcium have been identified (Annicchiarico and Thami-Alami, 2012). The primary source of phosphate fertilizer (mined rock phosphate) is considered likely to be depleted within 40 years, emphasizing the importance of sustainable P management. When available P is limiting, lupins form specialized cluster root structures and/or release P-mobilizing carboxylates that free it from insoluble forms (Lambers et al., 2012), which could be exploited more widely to reduce the need for P supplementation.

To combine high protein quality, adaptation to environmental stresses and high yield, a suite of modern techniques is required. Extensive research in Australia has provided valuable information on the genetics of narrow-leafed lupin, and its genome sequence is nearly complete. Genomic selection is a particularly promising tool for improving complex, quantitatively inherited traits (Heffner et al., 2009). Combining phenotyping and genotyping data of the population into a model enables breeding values for each marker to be estimated. Genotyping-by-sequencing (GBS) can provide thousands of single nucleotide polymorphism (SNP) markers at much lower cost than earlier techniques (Elshire et al., 2011), but should be optimized for individual crops. GBS can help to identify and establish valuable genetic markers needed for genomic selection of each lupin species, and ultimately, to select cultivars adapted to the wide variety of European soils and climates, and to allow productive cultivation on poor and marginal lands.

Lupins form root nodules in which biological nitrogen fixation (BNF) takes place in symbiosis with compatible soil bacteria of the genus *Bradyrhizobium*. In general, European lupins that grow in acid soils can establish effective symbiosis with bradyrhizobia that are usually present in these soil, although they may be absent from neutral or alkaline soils. Where indigenous rhizobia are inadequate, inoculants boost BNF, improving grain yield and often its protein quality. BNF is strongly related to the physiological state of the host plant, so that environmental stresses not only have a detrimental effect on metabolism, growth and development of the plant, but also affect symbiosis. The *Lupinus-Bradyrhizobium* symbiosis has been described as being relatively tolerant to abiotic stresses (Fernández-Pascual et al., 2007). Rhizobial strain and lupin genotype interactions influence nodulation score, nitrogen fixation and plant growth. Specific *Bradyrhizobium* strains should be developed together with stress-tolerant lupin genotypes, to promote lupin as a successful component of sustainable crop production systems.

To use lupin as a source of high quality and healthy proteins for food, several quality traits are worth extra attention: in particular, alkaloid content, which must be maintained as low as possible, and the conglutin-γ protein fraction, which is of interest to control insulin resistance and diabetes (Terruzzi et al., 2011). The oil content of white lupin is 8–14% and its nutritional quality is excellent (Boschin et al., 2008). If this trait is further improved by breeding, it would increase the economic sustainability of the crop by making it dual-purpose for protein and oil, like soya bean. Iron deficiency is one of the most common and widespread nutritional disorder worldwide. Traditional iron supplementation methods can have negative effects on the consumer, and are unavailable to many people. The Fe-rich protein ferritin is abundant in some legume seeds, including lupin (Strozycki et al., 2007), and may represent a safe way to increase dietary iron intake. Lupin proteins influence lipid and glucose metabolism, as well as blood pressure levels (Duranti and Morazzoni, 2011). Amino acid sequences that may be related to hypotensive and lipid-lowering activities have been detected in lupin proteins (Bettzieche et al., 2009), although the components responsible for these effects are still undetermined and the underlying mechanisms remain ill-defined. Another interesting aspect of lupin protein functionality is its possible effects on inflammatory processes and changes in the gut microbiome, which has a significant influence on several physiological parameters, including metabolism, nutrient absorption and immune function (Walsh et al., 2014).

Food processing affects structural features of proteins and peptides, exerting a strong impact on their techno-functional and bioactive properties. Some proteins display their activity through peptides that must be liberated upon digestion to achieve functionality, while other proteins, such as conglutin-γ, seem to be required in an intact form. The production of protein ingredients with improved nutritive, technological and health benefits presents the food industry with unique challenges. Technological approaches remain to be optimized to improve production, cost effectiveness, and sustainable and environmental feasibility of high-quality protein ingredients. The selection of pre-treatments, such as germination in mineral-fortified conditions (Frias et al., 2009), and the optimization of processing conditions to facilitate protein extraction and isolation, represent important technological targets, oriented toward sustainable and non-invasive techniques. Off-flavor production is an important constraint in legume flour milling, storage and use (Azarnia et al., 2011). Pre-treatment with electromagnetic microwave radiation provides potential advantages over conventional hydro-thermal pre-treatment, inactivating enzymes that cause off-flavor formation and enhancing seed brittleness, thereby assisting milling and fractionation with little effect on protein functionality, as has been demonstrated in oat (Keying et al., 2009). These techniques need to be properly adapted to lupin seeds. Different fractionation, isolation and purification techniques combined with pre-processing are used to obtain protein concentrates/isolates with targeted techno-functional properties. Wet fractionation techniques are conventionally used to obtain relatively pure protein isolates, although they are energy-intensive procedures requiring large amounts of water and they may affect protein functionality (Muranyi et al., 2013). Many processing conditions for protein isolation are well established, but they are amenable to improvement, as shown by the influence of various parameters on target properties of the protein preparation and on overall protein yield. Dry milling combined with air classification is an alternative strategy to wet extraction, and may provide ideal disentanglement with lower energy and water demands (Schutyser and van der Goot, 2011).

Protein isolates prepared by isoelectric precipitation exhibit excellent water absorption, emulsification and foam-forming activities (Rodríguez-Ambriz et al., 2005). In contrast, legume protein isolates prepared by salt-induced extraction and dilution-precipitation with water have outstanding oil-binding capacity, emulsion stability and specific viscoelasticity, which affect their rheological properties, e.g., for bakery products (Marco and Rosell, 2008). Hence, the techno-functional properties of lupin proteins must be examined to optimize processing conditions and select suitable processing schemes, such as protein modification by microstructuring, to obtain ingredients for different food applications. Protein hydrolysates containing bioactive peptides are of great interest for the design of functional foods and nutraceuticals. Proteolysis of lupin storage proteins confers added value and targets functional properties as well as decreasing allergenicity (Clemente, 2000), and is commonly achieved by enzymatic treatments and fermentation. Recently developed high hydrostatic pressure and ultrasound-assisted methods increase the hydrolytic efficiency of enzymes (Zhang et al., 2012; Garcia-Mora et al., 2015). Sensory qualities and storage stability are key parameters to be taken into account when introducing lupin ingredients into the food industry. For example, neutral taste should be assured and maintained over a certain period in storage. Therefore, endogenous enzyme activities must be characterized to ensure the quality of the protein produced and to monitor storage stability. Lupin fiber could help to stabilize lupin protein based structures, as shown for other plant fiber materials (Laine et al., 2011). Accompanying components affect the techno-functional properties of the final protein products, so this has to be considered.

Technological challenges to optimize the production and processing of lupin protein relate to the integration of the process into a holistic concept that considers oil and fiber recovery, use of co-products for animal feed, bio-fuel (Pakarinen et al., 2011), or soil amendments, or even water recycling. After protein extraction, the large amount of dietary fiber (up to 40% of seed mass) has a potential role in functional foods. Innovative, cost-effective and environmentally friendly technologies need to be explored for fiber purification and fractionation. Lupin oil is an attractive product because of its balanced fatty acid composition and its content of bioactive lipids (Kalogeropoulos et al., 2010). Within the context of co-product utilization, animal feed is one of the main contributors to overall profitability.

The safety aspects of lupin ingredients include the formation of biogenic amines and the presence of allergens. Lupin allergy is still quite rare, but cross-reactivity between peanut and lupin exists (Fiocchi et al., 2009), which has led to the inclusion of lupins in the allergen list of the EU directive on labeling (EU Directive 2006/142/EC). Conglutins-α, -β, -γ, and -δ are candidate allergens in lupin, of which conglutin-α has strong allergenicity (Holden et al., 2008). Considering the severity of allergic reaction to peanut, the cross-reactivity of new lupin derivatives needs to be carefully assessed, and commercially exploited lupin products properly labeled, to minimize the danger for potential allergic consumers.

Lupin ingredients are already used in foods in Europe but their use is much less common than that of soya bean or pea ingredients, despite their beneficial properties. Lupin ingredients (flour and protein isolates and concentrates) are mainly used in bakery and gluten-free products, albeit as minor components (<5% of the ingredients). Nevertheless, new products containing lupin ingredients enter the European market each year (Mintel GNPD, http://www.mintel.com/global-new-products-database). Given its high protein content, lupin flour is considered an excellent raw material to supplement different food products (Pollard et al., 2002) and can be used as an egg substitute in cakes, pancakes, biscuits, pasta, or bread (Dervas et al., 1999). The rich yellow color of lupin flour and some protein concentrates has considerable appeal and can be of value in pasta or noodle dishes (Doxastakis et al., 2002), yet it can be easily eliminated when necessary (Guémes-Vera et al., 2008). Lupin flour can be incorporated into wheat flour to improve the nutritional value of the final products with little or no detriment in product sensory quality, and lupin fiber can also be used as a source of dietary fiber (Clark and Johnson, 2002). In general, the addition of up to 10% lupin flour improves water binding, texture, shelf-life and aroma of bread, although the mixing time and dough stability decrease as the proportion of lupin flour is increased (Doxastakis et al., 2002; Pollard et al., 2002).

While European consumers have a positive opinion on plant protein consumption, many do not know that lupin is a plant protein source comparable to soya bean. Among the main trends in the current European food market are naturalness; environmentally friendly; vegetarian alternative to meat, milk or eggs; and health properties (Mintel GNPD). The development of novel lupin-based foods responding to these desiderata should probably focus first on replacement of animal products (meat alternative, vegetarian spreads, dessert creams, ice-cream, and vegetable drinks). Gluten-free products are often lower in protein than standard goods, and lupin-based goods (bakery products, pasta, breakfast cereals) would restore or even increase the protein content. Further targets include high-protein food products with excellent sensory properties (sausages, pasta, snacks, drinks, bread). Specific consumer groups can be addressed with suitable nutritional values, taste and texture. The major international nutraceutical markets (EU, USA, Japan) are expected to grow in the near future, with major areas of interest being chronic degenerative cardiovascular diseases, type-2 diabetes and neuroprotection. Despite their health-promoting effects (Duranti and Morazzoni, 2011; Terruzzi et al., 2011), lupin ingredients are scarcely used in probiotics and nutraceuticals, as only a few lupin-containing food supplements and products with health claims are present in Europe (Mintel GNPD). The European Food Safety Authority (EFSA) currently allows no health claim about lupin ingredients. If proper clinical trials demonstrate a preventive role of lupin-based foods toward ailments such diabetes, cardiovascular diseases, metabolic syndrome, and obesity, these health-promoting features may confer an important advantage over other protein crops.

Lupin production in Europe declined steadily during the second half of the 20th century (Preissel et al., 2015), mainly because of low productivity driven by seasonal variability (Cernay et al., 2015), the low price of lupin grain (de Visser et al., 2014), and EU policies favoring the importation of soya bean. Concern about the sustainability and dependence of non-European protein sources, as well as potential environmental advantages, have re-established interest in lupins and other legume crops, and production has increased since 2003 (Figure 1). To establish lupin-based products as serious alternatives, the supply chain of these products must be assessed in terms of production costs, yields and quality, as well as factors influencing the acceptance of lupin-based products by European consumers (de Visser et al., 2014). The complete supply chain has to be addressed, analyzing the factors contributing to low lupin production by European farmers, and the conditions necessary to increase supply and make lupin a viable alternative to existing crops. The potential benefits of lupins at the farm level include N fixation with positive direct and indirect effects for subsequent crop yields, lower phosphorus requirements, reduced soil loss, and biodiversity benefits (Nemecek et al., 2008). Value-chain characteristics to be considered comprise agricultural production in terms of cost factors and yield effects, agricultural policies driving farmers’ decisions, production quantities and qualities demanded by processing companies, sustainability impacts, and factors influencing the acceptance of lupin products by European consumers. Life-cycle assessment methods (LCA) and participatory impact assessment techniques should be combined in a complementary manner. An in-depth investigation of the causes and driving forces leading to the current production and consumption situation of lupins in Europe would indicate which characteristics lupins have to fulfill and how framework situations need to be changed (e.g., agricultural production conditions, level of substitution in end-products) to make lupins into profitable protein crops.

The rising global demand for meat, dairy and fish products for human consumption is recognized as unsustainable, owing to the high environmental impact of animal production. Furthermore, diseases associated with excessive consumption of animal products are increasing in frequency. The world population continues to grow, so a trend toward diets containing more plant protein seems not just strongly recommended, but inevitable, and it would be wise to anticipate and direct this trend by adopting knowledge-based strategies. To best achieve this objective, advances in virtually all phases of the supply chain are needed. Lupins should be established as fundamental crops in the various agro-climatic zones and marginal lands of Europe, and their yields and adaptation genetically improved to ensure a continuous supply of quality grain. The combination of their low needs for fertilizer N and P with their exploitation of poor, degraded, stress-affected or contaminated soils to safely produce protein-rich crops could contribute to socio-economic development and environmental sustainability. Sustainable, innovative, and cost-efficient processing methods to produce high-protein ingredients should be devised to guarantee the socio-economic value of these crops. Reformulating traditional foods, rather than developing totally novel ones, may be a more effective strategy to capture the imagination of the European consumer, and lead to desirable changes in overall diet. There is huge potential market demand for lupin-based products, with niches in growing sectors, such as vegetarians, vegans, and people with intolerance or allergy to gluten, soya, milk, or egg. The incorporation of lupin ingredients as a source of protein for human consumption depends largely upon their nutritional quality, but also on their ability to be used as, or incorporated into, foods that will be readily consumed. There are European enterprises with a strong background and wealth of expertise on market analysis, consumer acceptance issues, legal issues and marketing tools, which are required to develop new and successful lupin products that constitute a serious European alternative to the prevailing soya bean-derived food monopoly.

## Funding

This work was supported by a grant from MINECO (AGL2013-40758-R).

### Conflict of Interest Statement

The authors declare that the research was conducted in the absence of any commercial or financial relationships that could be construed as a potential conflict of interest.
